# Childhood-onset parkinsonism, dystonia and severe muscle wasting in a Sudanese girl caused by a novel homozygous DnaJ heat shock protein family (heat shock protein 40) member C6 frameshift variant, with an incidental pathogenic breast cancer 1 gene secondary finding: a case report

**DOI:** 10.1186/s13256-026-06351-x

**Published:** 2026-07-22

**Authors:** Khabab Abbasher Hussien Mohamed Ahmed, Razan Kamal Yousif Ahmed

**Affiliations:** 1 Faculty of Medicine, University of Khartoum, 11111 Khartoum, Sudan; 2 Department of Pathophysiology, School of Medicine, St. George’s University, St. George’s, Grenada; 3 Faculty of Medicine, Al Mughtaribeen University, 11111 Khartoum, Sudan

**Keywords:** *DNAJC6*, Juvenile parkinsonism, Dystonia, *BRCA1*, Whole-exome sequencing, Secondary findings, Conversion disorder, Oculogyric crises, Consanguinity, Case report

## Abstract

**Background:**

*DNAJC6*-related juvenile parkinsonism is a rare autosomal recessive disorder of auxilin, a co-chaperone essential for clathrin-mediated synaptic vesicle recycling. Fewer than 50 kindreds have been reported worldwide, and the condition is easily missed when first-line investigations come back normal. We describe a Sudanese adolescent in whom a previously unreported homozygous *DNAJC6* frameshift variant was identified only after a prolonged diagnostic journey that included a period during which her illness was mistakenly attributed to a functional neurological disorder. Clinical whole-exome sequencing additionally returned an incidental medically actionable *BRCA1* secondary finding.

**Case presentation:**

A 13-year-old Sudanese girl, the eldest child of first-cousin parents, was born preterm and ventilated neonatally for severe respiratory distress. Her development was normal until age five, when an isolated upper-limb tremor first appeared. Over the next 8 years her condition progressed into severe juvenile parkinsonism: bilateral rest-and-action tremor, rigidity, bradykinesia, generalized dystonia with prominent oromandibular involvement, dysphagia, profound cachexia, cognitive and behavioral decline, sleep and autonomic disturbance, episodic oculogyric crises and three discrete seizures. By approximately 11 she was wheelchair-dependent. Earlier, unrevealing investigations had led to a psychiatric diagnosis of functional neurological disorder, and the dopamine antagonists prescribed on that basis worsened her condition. Whole-exome sequencing later identified a novel homozygous *DNAJC6* frameshift deletion (NM_001256864.2:c.1480del; p.Glu494ArgfsTer12), absent from population databases, alongside a heterozygous pathogenic *BRCA1* deletion reported as an actionable secondary finding. Levodopa produced partial motor benefit, complicated by on-state dyskinesias, visual hallucinations and excessive daytime somnolence. Subsequent up-titration of carbidopa/levodopa to approximately 5-hourly dosing produced a further, clinically evident improvement in gait and fine-motor function.

**Conclusions:**

This case adds a previously unreported allele to the *DNAJC6* catalog and characterizes the severe end of its phenotype, where corticospinal signs and life-threatening cachexia accompany the more familiar extrapyramidal features. It illustrates the harm that follows premature labeling of a progressive organic movement disorder as functional, especially when dopamine antagonists are then prescribed. The accompanying pathogenic *BRCA1* secondary finding shows the dual diagnostic yield that clinical whole-exome sequencing can deliver. Early genetic investigation in childhood-onset movement disorders, particularly with consanguinity, should be the rule, and cascade pathways for actionable secondary findings embedded in routine practice.

**Supplementary Information:**

The online version contains supplementary material available at https://doi.org/10.1186/s13256-026-06351-x.

## Background

Most Parkinson disease declares itself late in life. A smaller, expanding group of monogenic forms presents in childhood, adolescence or early adulthood, and these early-onset cases often look quite different from the classical adult picture—a divergence that contributes to their long diagnostic odysseys [1].

*DNAJC6*-related Parkinson disease sits within this group. The condition was first described in 2012 in two consanguineous Palestinian families, in whom affected children developed juvenile parkinsonism alongside intellectual disability and seizures [2]. The gene encodes auxilin, a neuron-specific co-chaperone that works in obligate tandem with heat shock cognate protein 70 to disassemble clathrin-coated vesicles at the synaptic terminal; without that step, vesicle recycling stalls [3]. Loss of auxilin therefore causes clathrin-coated vesicles to accumulate, depletes the readily releasable synaptic vesicle pool, and produces selective dopaminergic neurodegeneration. The catalog of *DNAJC6* variants has since expanded to include truncating, splice-site and missense alleles, and the phenotypic range now stretches from severe juvenile-onset disease (with dystonia, oculogyric crises, seizures and cognitive decline) to milder early-adult forms that resemble classical Parkinson disease [4, 5]. The total number of reported kindreds nonetheless remains small.

Children with progressive movement disorders frequently endure protracted diagnostic journeys when first-line investigations return normal. A familiar pitfall, with consequences that can be severe, is the premature attribution of organic disease to a functional neurological disorder [6, 7]. Prescribing dopamine receptor antagonists to a patient with unrecognized dopaminergic neurodegeneration adds pharmacological injury to diagnostic error, yet the harm is too often overlooked. We describe a patient in whom precisely that sequence unfolded over 8 years before whole-exome sequencing identified a previously unreported *DNAJC6* variant together with an incidental pathogenic *BRCA1* variant of independent clinical significance.

## Case presentation

### Demographics and family background

Our patient is a 13-year-old Sudanese girl of African ethnicity, presently resident in the Middle East. She is the eldest of her parents’ children. Her parents are first cousins. This kind of union confers substantial a priori risk for an autosomal recessive condition [8], a consideration that, on reflection, was not pursued early enough in her diagnostic journey. The family lives in modest socioeconomic circumstances, and she had to abandon formal schooling at grade four as her illness advanced.

Her maternal uncle has a documented diagnosis of epilepsy, and her maternal grandmother had been diagnosed with ovarian cancer—a piece of family history that took on new significance when the *BRCA1* secondary finding was reported. Her younger brother carries a diagnosis of autism spectrum disorder. No similar progressive neurological disorders have been reported elsewhere in the family.

### Perinatal and developmental history

Conception followed roughly 3 years of parental subfertility, during which a hysterosalpingography had been performed; the pregnancy itself was spontaneous rather than assisted. She was delivered by cesarean section at approximately 7 months’ gestation (around 31 weeks) with a birth weight of 900 g [9]. Her neonatal course was complicated by severe respiratory distress requiring mechanical ventilation, followed by a prolonged stay in the neonatal intensive care unit.

Despite that difficult start, her early milestones came on time across the gross-motor, fine-motor, language and social domains. There was no developmental delay or regression at any point before her neurological illness began, and her cognitive and functional status remained essentially normal until around 11 years of age.

### Symptom evolution

Her first neurological symptom appeared at about 5 years of age, when her family noticed a mild, intermittent tremor in her upper limbs. The tremor was predominantly action-induced. At that stage there was no rigidity, gait disturbance, weakness, dystonia or cognitive impairment, and her schoolwork and daily activities were unaffected.

Over the next several years the tremor worsened, became bilateral, and acquired both resting and action components. Rigidity and bradykinesia developed alongside a generalized slowing of voluntary movement. By mid-childhood her gait had become recognizably parkinsonian—shuffling, with reduced arm swing, freezing episodes and recurrent falls. By approximately 11 years of age she had lost independent ambulation entirely and was wheelchair-dependent.

Dystonia emerged in parallel. It progressively involved the neck, axial musculature, upper and lower limbs (most prominently the lower-limb adductors), and the oromandibular muscles, producing abnormal posturing, dysarthria and feeding difficulties. As the disease advanced, further features appeared: cognitive decline, behavioral changes, sleep disturbance, chronic constipation, urinary incontinence, episodic oculogyric crises, and three discrete clinical seizures in the later phase of her illness. There was no history of central nervous system infection, acute encephalopathy, toxin exposure or traumatic brain injury at any point.

### Diagnostic pathway and the misdiagnosis as functional neurological disorder

When her condition deteriorated in mid-childhood, the family first consulted a general pediatrician, who requested thyroid function tests; these returned within normal limits. About 2 months later she was referred to a neurologist, who in turn referred her to a psychiatry service. A diagnosis of functional neurological disorder (conversion disorder) was made.

A multidrug regimen followed: fluoxetine, carbamazepine 200 mg twice daily, amisulpride 50 mg twice daily, trihexyphenidyl 2 mg twice daily, risperidone 2.5 mg twice daily and carbidopa/levodopa. A short-lived partial improvement gave way to progressive deterioration, and all medications were eventually discontinued pending re-evaluation. It was the deterioration on treatment—rather than the failure of the diagnosis to fit—that ultimately prompted comprehensive genetic assessment.

### General examination

**Fig. 1:**
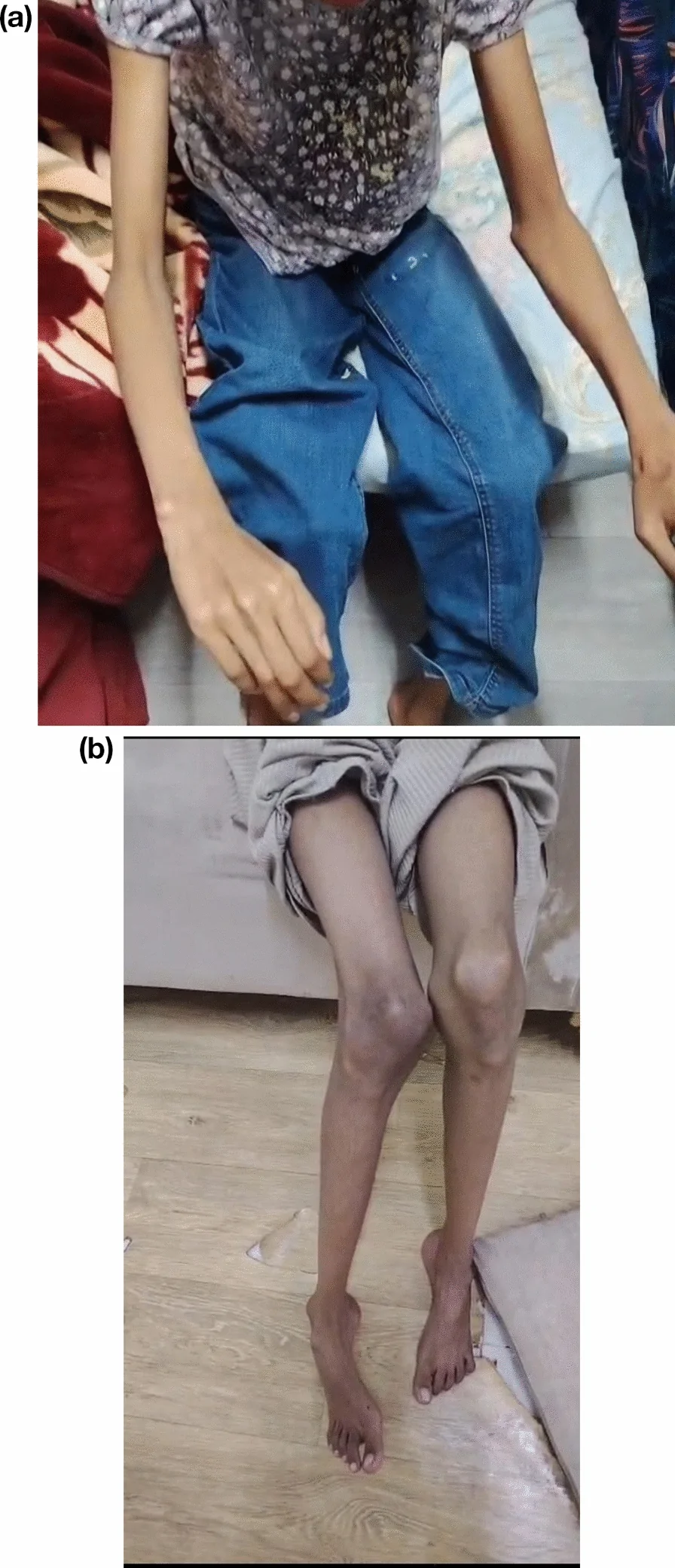
Clinical photographs demonstrating the marked degree of muscle wasting at specialist assessment. **A** Upper limbs showing profound generalized wasting with prominent loss of bulk in the deltoid, biceps, triceps and small muscles of the hand, consistent with severe cachexia. **B** Lower limbs showing equally striking generalized wasting with prominent loss of bulk in the quadriceps, hamstrings and calf compartments. Both photographs were obtained with written informed consent from the patient’s legal guardian; no facial features are shown and identifying features have been excluded

At specialist assessment she was chronically ill, profoundly cachectic and underweight, with generalized muscle wasting across all limb girdles and the paraspinal muscles (Fig. 1A and B). She was conscious, alert and cooperative, with dysarthric speech; higher mental function had initially been preserved but was by now in progressive decline, although comprehension remained relatively intact. She was wheelchair-dependent and needed support for transfers and ambulation. There were no dysmorphic features, no neurocutaneous stigmata, and no pallor, cyanosis, clubbing, jaundice, edema or significant lymphadenopathy; hydration was fair and vital signs stable.

### Neurological examination

Cranial nerves: pupils were equal and reactive and visual function grossly intact. Extraocular movements were full, though intermittent oculogyric crises had been documented during the disease course. Facial symmetry, hearing and the gag reflex were normal, and the tongue was central without wasting or fasciculation.

**Fig. 2:**
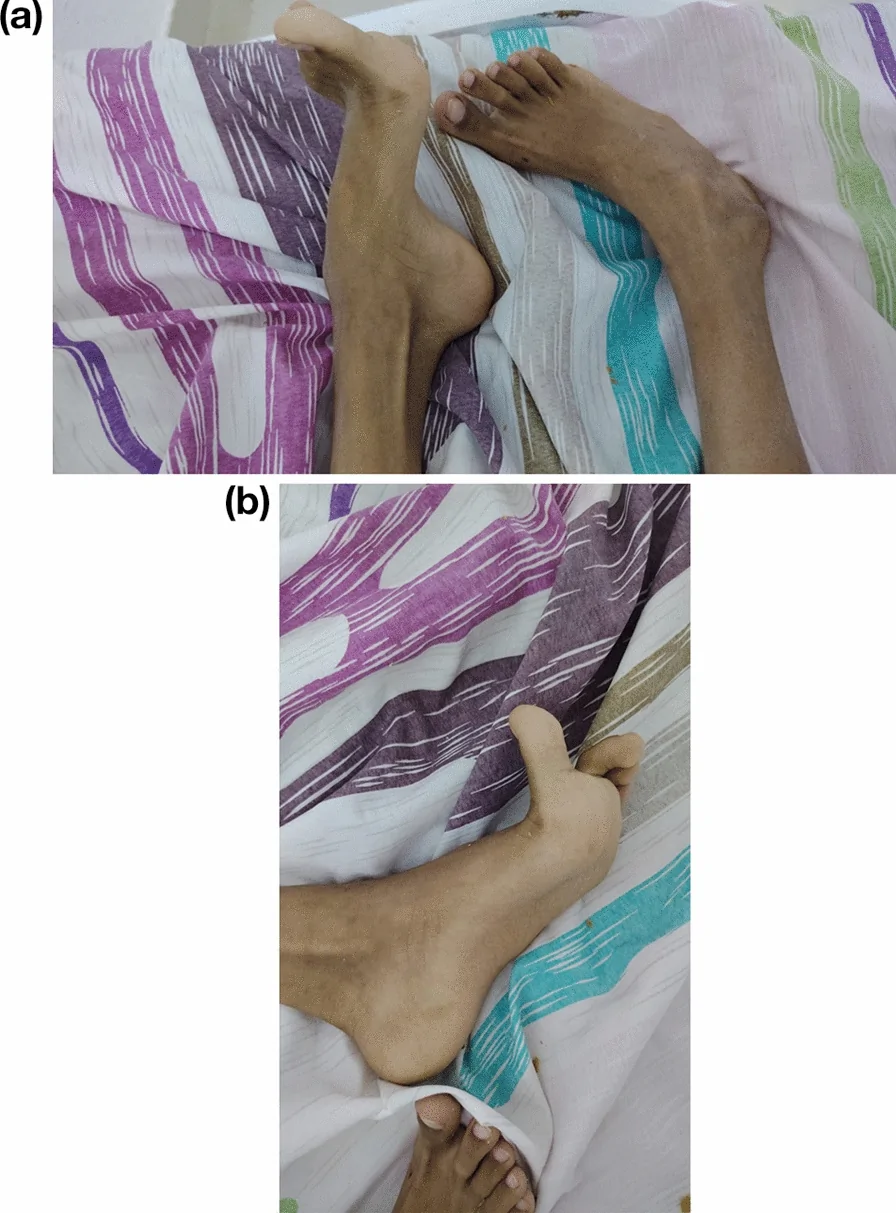
Clinical photographs of the feet demonstrating bilateral pes cavus. **A** Both feet showing a high medial longitudinal arch with associated clawing of the toes, consistent with pes cavus. **B** Lateral view of the foot showing the pronounced high arch. Both photographs were obtained with written informed consent from the patient’s legal guardian; no facial features are shown and identifying features have been excluded

Motor-tone: tone was increased in all four limbs, more so in the legs and on the right, with a lead-pipe rigidity that exceeded any spastic component; both Achilles tendons were tight, and both feet showed a fixed high-arched (pes cavus) deformity with associated toe clawing (Fig. 2A and B). Prominent dystonia involved the axial muscles, neck, upper limbs and lower-limb adductors, accompanied by profound bradykinesia and a bilateral mixed rest-and-action tremor.

Motor-power: detailed Medical Research Council motor power grading is set out in Table 1. In brief, upper-limb power ranged from 3/5 to 4/5 and lower-limb power from 3/5 to 4 + /5, with wrist movements and ankle dorsiflexion/plantarflexion the weakest at 3/5.

**Table 1:** Detailed Medical Research Council motor power grading at specialist assessment

Muscle group/movement	Right upper limb	Left upper limb	Right lower limb	Left lower limb	Normal
Elbow flexion	4/5	3 + /5	–	–	5/5
Elbow extension	4/5	3/5	–	–	5/5
Wrist flexion	3/5	3/5	–	–	5/5
Wrist extension	3/5	3 + /5	–	–	5/5
Shoulder abduction	3 + /5	3/5	–	–	5/5
Shoulder adduction	4/5	3/5	–	–	5/5
Hand grip/power	3 + /5	4/5	–	–	5/5
Hip flexion	–	–	4/5	4/5	5/5
Hip extension	–	–	4/5	4/5	5/5
Knee flexion	–	–	3/5	4/5	5/5
Knee extension	–	–	4 + /5	4 + /5	5/5
Ankle flexion (dorsi-)	–	–	4/5	3/5	5/5
Ankle extension (plantar-)	–	–	3/5	3/5	5/5

Medical Research Council scale: 0 = no contraction; 1 = flicker; 2 = movement with gravity eliminated; 3 = movement against gravity; 4 = movement against reduced resistance; 5 = normal power. Cerebellar testing was limited by tremor, rigidity, dystonia and bradykinesia. Deep tendon reflexes were exaggerated bilaterally in the lower limbs, and plantar responses were extensor bilaterally

Reflexes and sensation: deep tendon reflexes were exaggerated bilaterally in the lower limbs and plantar responses were extensor on both sides, pointing to corticospinal involvement alongside the extrapyramidal syndrome. Sensation was grossly intact to light touch and pinprick, with no sensory level or peripheral deficit.

Coordination: reliable cerebellar testing was not achievable given the severe rigidity, bradykinesia, dystonia and weakness; no definite cerebellar signs or choreiform movements were seen.

Involuntary movements: she showed a generalized tremor of mixed rest-and-action character, with superimposed dystonic posturing affecting particularly the distal upper and lower limbs (Additional file 1 & Additional file 2: Video 1 & Video 2). On-state choreiform dyskinesias attributable to levodopa were also intermittently apparent.

Gait: standing and walking required support. When attempted, gait was severely abnormal and showed a spastic-parkinsonian pattern with reduced arm swing, freezing, postural instability, right-sided predominance and recurrent unsteadiness (Additional file 3: Video 3).

## Investigations

Laboratory studies: thyroid function tests, requested at her initial pediatric assessment, were normal. A complete blood count showed mildly elevated hematocrit (45.9%; reference 35–45%), with reduced mean platelet volume (7.1 fL; reference 8–12 fL) and reduced platelet distribution width (8.8; reference 10–18); other indices were normal. Iron studies showed mildly elevated total serum iron (110 µg/dL; reference 30–109 µg/dL) and ferritin (113 ng/mL; reference 13–84 ng/mL). Serum magnesium and vitamin B1 were both within reference ranges, the latter ruling out nutritional thiamine deficiency.

Liver function: liver function tests obtained earlier in her disease course had been markedly deranged—transaminases, alkaline phosphatase, gamma-glutamyl transferase, total and direct bilirubin, and globulin were all elevated, with low albumin. That profile raised an immediate concern for Wilson disease. Repeat testing approximately 10 months later showed complete normalization across all parameters—a course inconsistent with the progressive hepatic deterioration expected in untreated Wilson disease, and more plausibly attributable to medication-related hepatotoxicity from the prior polypharmacy regimen, a transient intercurrent hepatitis or a self-limiting hepatic event. The complete investigation summary appears in Table 2.

**Table 2:** Summary of laboratory, radiological and genetic investigations

Investigation	Result	Reference range	Interpretation	Source
Initial pediatric workup				
Thyroid function tests	Within normal limits	–	Normal; excluded thyroid-related tremor	Initial workup
Hematology and biochemistry				
Hemoglobin	15.5 g/dL	11.7–15.5 g/dL	Normal (upper limit)	Lab
**Hematocrit**	**45.9% (high)**	**35–45%**	**Mildly elevated**	**Lab**
**Mean platelet volume**	**7.1 fL (low)**	**8–12 fL**	**Reduced**	**Lab**
** Platelet distribution width**	**8.8 (low)**	**10–18**	**Reduced**	**Lab**
White cell count and differential	Normal	–	Normal	Lab
**Total serum iron**	**110 µg/dL (high)**	**30–109 µg/dL**	**Mildly elevated**	**Lab**
**Serum ferritin**	**113 ng/mL (high)**	**13–84 ng/mL**	**Mildly elevated**	**Lab**
Serum magnesium	2.26 mg/dL	1.62–2.52 mg/dL	Normal	Lab
Vitamin B1 (thiamine)	47.7 µg/L	28–85 µg/L	Normal	Lab
Liver function—earlier in disease course (markedly abnormal)				
**Alanine aminotransferase**	**462 U/L (high)**	**5–55 U/L**	**Markedly elevated**	**Historical**
**Aspartate aminotransferase**	**232 U/L (high)**	**5–48 U/L**	**Markedly elevated**	**Historical**
**Alkaline phosphatase**	**586 U/L (high)**	**15–218 U/L**	**Markedly elevated**	**Historical**
**Gamma-glutamyl transferase**	**744 U/L (high)**	**1–55 U/L**	**Markedly elevated**	**Historical**
**Total bilirubin**	**17.87 mg/dL (high)**	**0.15–1.2 mg/dL**	**Markedly elevated**	**Historical**
**Direct bilirubin**	**13.50 mg/dL (high)**	**0–0.3 mg/dL**	**Markedly elevated**	**Historical**
**Albumin**	**3.18 g/dL (low)**	**3.5–5 g/dL**	**Low**	**Historical**
**Globulin**	**4.13 g/dL (high)**	**1.5–3.5 g/dL**	**Elevated**	**Historical**
Liver function—repeat testing (fully normalized)				
Alanine aminotransferase	20.01 U/L	≤ 55 U/L	Normal	Lab
Aspartate aminotransferase	32.21 U/L	5–34 U/L	Normal	Lab
Alkaline phosphatase/GGT	99.54/17.65 U/L	Within range	Normal	Lab
Total/direct bilirubin	5.58/1.25 µmol/L	Within range	Normal	Lab
Albumin/total protein	4.62 g/dL/65.13 g/L	Within range	Normal	Lab
Neuroimaging and electrophysiology				
Brain magnetic resonance imaging	Normal—no basal-ganglia, white-matter, structural or vascular abnormality	–	Excluded structural lesions, leukodystrophy and brain iron accumulation	Radiology
Brain computed tomography	Entirely normal	–	Excluded gross structural abnormality	Radiology
Electroencephalogram	Normal background; no epileptiform activity	–	No interictal abnormality; three clinical seizures subsequently occurred	Clinical
Repeat electroencephalogram (awake + sleep)	Planned	–	To re-evaluate following clinical seizures, accounting for drug effects	–
Creatine kinase/urine myoglobin	Pending	–	Ordered to evaluate myopathic contribution to wasting	–
Scoliosis series imaging	Planned	–	Indicated given movement-disorder severity	–
Whole-exome sequencing (Additional file 4)				
***DNAJC6* (primary finding)—autosomal recessive**	**Homozygous pathogenic frameshift NM_001256864.2:c.1480del p.Glu494ArgfsTer12**	**Absent from gnomAD; novel**	**Pathogenic; *DNAJC6*-related juvenile parkinsonism**	**Add. file 4**
***BRCA1* (secondary finding)—autosomal dominant**	**Heterozygous pathogenic deletion NM_007294.4:c.4041_4042del p.Gly1348AsnfsTer7**	**Established pathogenic**	**Pathogenic; ACMG-actionable; HBOC syndrome**	**Add. file 4**

Entries in bold indicate clinically significant findings — laboratory values that fall outside the reference range and the pathogenic genetic variants identified. *ACMG* American College of Medical Genetics and Genomics; *BRCA1* breast cancer 1 gene; *c.* coding DNA reference sequence (HGVS); *DNAJC6* DnaJ heat shock protein family (heat shock protein 40) member C6 gene; *fL* femtoliter; *g/dL* grams per deciliter; *g/L* grams per liter; *GGT* gamma-glutamyl transferase; *gnomAD* Genome Aggregation Database; *HBOC* hereditary breast and ovarian cancer; *mg/dL* milligrams per deciliter; *ng/mL* nanograms per milliliter; *NM_* RefSeq transcript reference sequence; *p.* protein reference sequence (HGVS); *U/L* units per liter; *μg/dL* micrograms per deciliter; *μmol/L* micromoles per liter

Imaging and electrophysiology: an electroencephalogram performed during the diagnostic workup was reported as normal, with no epileptiform discharges, no paroxysmal activity and no periodic complexes—although three discrete clinical seizures subsequently occurred. Brain magnetic resonance imaging was entirely normal: ventricular system unremarkable, no gray- or white-matter signal abnormality, normal brainstem and cerebellar hemispheres, and no demyelination, hemorrhage, infarction or cortical dysplasia. There was no basal-ganglia signal abnormality, no leukodystrophic change and no other structural neurodegenerative finding. Brain computed tomography was also entirely normal. The lack of structural neuroimaging abnormality fits with published *DNAJC6* reports, in which conventional imaging is commonly normal in earlier disease phases [2, 5].

Whole-exome sequencing: whole-exome sequencing was carried out at an accredited clinical genetics laboratory. Genomic DNA was extracted from a dried blood-spot specimen and sequenced on a NovaSeq X platform (Illumina, San Diego, CA, USA) at a mean depth-of-coverage of 204.87 × , with 98.70% of targeted bases covered at ≥ 20 × . Variants were prioritized through an automated bioinformatic pipeline [10] and classified in line with the American College of Medical Genetics and Genomics/Association for Molecular Pathology guidelines [11]. The full whole-exome sequencing report is provided as Additional file 4.

Primary finding—*DNAJC6* (autosomal recessive): a homozygous frameshift deletion was identified at genomic position 1-65392440-CG-C (GRCh38), designated NM_001256864.2:c.1480del, predicting the protein change p.Glu494ArgfsTer12. The single-nucleotide deletion shifts the reading frame from codon 494 and introduces a premature stop codon twelve codons downstream. The variant is therefore predicted either to trigger nonsense-mediated mRNA decay or to yield a severely truncated auxilin protein lacking the C-terminal DnaJ domain that is essential for clathrin disassembly. It is absent from the gnomAD v4.1.0 dataset, has not previously been reported in any clinical variant repository or in the published literature, and was classified as pathogenic under American College of Medical Genetics and Genomics criteria [11].

Secondary finding—*BRCA1* (autosomal dominant): a medically actionable secondary finding was reported in parallel: a heterozygous pathogenic deletion at NM_007294.4:c.4041_4042del (p.Gly1348AsnfsTer7), classified as pathogenic and associated with hereditary breast and ovarian cancer syndrome. *BRCA1* sits on the American College of Medical Genetics and Genomics list of medically actionable genes for which reporting is mandated regardless of patient age or primary indication [12]. With ovarian cancer documented in the maternal grandmother, cascade testing of at-risk female relatives was strongly indicated.

## Differential diagnosis

Before genetic confirmation, several conditions were weighed: Wilson disease, Charcot–Marie–Tooth disease, Rett syndrome, subacute sclerosing panencephalitis, other neurodegenerative and metabolic disorders (particularly neurodegeneration with brain iron accumulation), and autoimmune encephalitis.

Wilson disease was our leading metabolic concern, given the combination of an early-onset movement disorder with prominent dystonia and the markedly abnormal liver enzymes documented earlier in her course [13]. The spontaneous normalization of her liver function tests on repeat testing—a pattern that does not fit untreated Wilson disease, which would be expected to deteriorate progressively—together with the eventual molecular diagnosis, effectively excluded it. A slit-lamp examination for Kayser–Fleischer rings was unremarkable.

Charcot–Marie–Tooth disease was considered because of the distal-predominant weakness on examination (most marked at the wrists and ankles), bilateral tight Achilles tendons, pes cavus (Fig. 2A and B) and severe distal muscle wasting [14]. The central features of her presentation—tremor, rigidity, generalized dystonia, oromandibular involvement, dysphagia and a hypokinetic-rigid gait—are not features of Charcot–Marie–Tooth disease, however, and the molecular identification of the *DNAJC6* variant excluded it.

Rett syndrome was considered on the basis of her sex and the repetitive involuntary movements, but the absence of the characteristic developmental regression pattern and the preservation of purposeful hand use argued conclusively against it. Subacute sclerosing panencephalitis was briefly entertained because of her progressive multisystem neurological course; the absence of the pathognomonic periodic high-voltage sharp-wave complexes on electroencephalography made it very unlikely [15]. Within the broader category of neurodegenerative disorders, neurodegeneration with brain iron accumulation deserved specific consideration; the normal brain magnetic resonance imaging—including the absence of basal-ganglia iron deposition or an “eye-of-the-tiger” sign—argued against this group of conditions [16]. Autoimmune encephalitis was considered given the subacute multi-feature presentation, but a fully explanatory monogenic diagnosis made an additional autoimmune contribution unlikely. Functional neurological disorder, although formally diagnosed at one stage, was ultimately incorrect; the molecular identification of biallelic *DNAJC6* loss-of-function variants provided a definitive organic explanation [6, 7].

## Treatment

Once her genetic diagnosis was made, she was commenced on carbidopa/levodopa, baclofen and olanzapine. Her initial response was partial: she regained some capacity for assisted ambulation, swallowing improved modestly, and tremor and rigidity were both reduced. Cachexia persisted, and she continued to require intensive nutritional support.

An expert specialist opinion subsequently recommended a revised regimen. Baclofen was continued at 5–10 mg per dose, titrated to response. A benzodiazepine (clonazepam or clobazam) was optimized, with a planned gradual taper if sustained stability could be achieved. Clonidine 25 µg at night was added to address sleep disturbance and provide adjunctive dystonia control. Levodopa dosing was adjusted in response to clinical motor assessment. Levetiracetam was introduced with gradual dose escalation to target any subclinical epileptiform activity. Olanzapine was scheduled for gradual taper and discontinuation: its dopamine receptor blockade is pharmacologically incompatible with the goals of dopaminergic supplementation [17].

Within days of these adjustments she developed increased muscle weakness, slowing of swallowing, decreased oral intake and daytime drowsiness lasting most of the day. The supervising clinician read these features as cumulative sedative and central nervous system depressant effects of the polypharmacy regimen and responded by reducing the clonazepam dose to one-and-a-half tablets once daily and the levetiracetam dose from twice-daily to once-daily administration. The medication-attributable symptoms resolved.

At her most recent specialist review, her active pharmacological regimen comprised carbidopa/levodopa 10/100 mg (initially four times daily and subsequently increased to approximately 5-hourly dosing; see “Outcome and follow-up”), together with clonazepam and baclofen. Clonidine and levetiracetam had been discontinued, and olanzapine had been successfully tapered off. Multidisciplinary care included structured physiotherapy (focused on stretching, contracture prevention, mobility support and positioning), occupational therapy (activities of daily living and adaptive equipment), and speech and swallowing evaluation (for dysarthria management, feeding strategies and aspiration risk). Because of the severity of her cachexia and the compounding role of oromandibular dystonia and dysphagia, nutritional rehabilitation was given the highest priority. A specialist dietitian designed a high-calorie, high-protein dietary plan modified for her swallowing capacity [18]. Nasogastric feeding was proposed as an interim nutritional bridge should oral intake again become inadequate, with percutaneous endoscopic gastrostomy held as a longer-term option.

## Outcome and follow-up

For much of her course, and despite the molecular diagnosis, dopaminergic therapy and substantial multidisciplinary input, her overall trajectory was poor. She had been wheelchair-dependent from approximately 11 years of age and required support for transfers and ambulation, and her cognitive status, initially preserved, showed progressive decline. She has not died and remains in active outpatient care. Return to formal schooling has not been possible, although home-based educational support has been started informally.

Earlier in her treatment course, a clinically important feature was the emergence of levodopa-induced complications. Despite progressive dose titration at that stage, her motor benefit had plateaued below the level normally expected in juvenile-onset levodopa-responsive parkinsonism, and classical dopaminergic side effects appeared in parallel—on-state dyskinesias, visual hallucinations and excessive daytime somnolence. The combination of incomplete benefit and dose-limiting complications initially constrained further escalation, and management shifted toward balancing symptom control against tolerability.

Her most recent follow-up, however, has been more encouraging. After carbidopa/levodopa was increased to 10/100 mg approximately every 5 hours (about five doses daily), the family reported—and serial home video confirmed—a clear improvement in motor function. She has regained the ability to stand and walk independently across a room without support (Additional file 5: Video 4), a marked change from her previous wheelchair dependence, and her distal hand function has improved sufficiently for her to apply nail polish unaided (Additional file 6: Video 5). Tremor and rigidity are further reduced, and she is able to take part in basic self-care with less assistance than before. These gains are partial and her disease remains severe, but they represent meaningful, patient-important functional improvement and underline the value of continued, careful dose optimization.

Parental genetic testing for the *DNAJC6* c.1480del variant has been initiated through the clinical genetics service to confirm the expected biparental heterozygous carrier status, with implications for any future pregnancies. Cascade *BRCA1* testing has been offered to her mother and to other at-risk female relatives, and a transitional oncological surveillance pathway is being arranged for her own progression into adulthood [19]. Multidisciplinary review at 3- to 6-monthly intervals is planned for at least the next 2 years.

## Discussion and conclusions

This case adds several pieces of useful information to the still-sparse *DNAJC6* literature [2, 4, 5]. Our patient carries a homozygous c.1480del frameshift deletion in *DNAJC6* that has not, to the best of our knowledge, been previously reported in any individual with Parkinson disease, in any clinical variant repository, or in the published literature, and is absent from large population reference databases. Variant novelty aside, her story brings into sharp focus several themes that recur in *DNAJC6*-related disease: a long diagnostic odyssey that begins in early childhood; the particular hazard of being mistakenly labeled with a functional neurological disorder; the multisystem character of advanced disease, which extends well beyond the extrapyramidal core to involve cognition, behavior, autonomic function and seizures; and the dual diagnostic yield—primary plus secondary findings—that clinical whole-exome sequencing can deliver [20].

### Molecular pathology of the c.1480del variant

The c.1480del deletion disrupts the coding sequence of *DNAJC6* at codon 494, within the C-terminal region of auxilin that encodes the DnaJ domain responsible for heat shock cognate protein 70 recruitment [3]. A premature stop codon located twelve codons downstream is predicted to trigger nonsense-mediated mRNA decay, with effective loss of functional auxilin protein. Even if a truncated transcript were to escape surveillance, the resulting protein would lack the structural machinery needed to catalyze clathrin disassembly. The downstream consequences—accumulation of clathrin-coated structures at dopaminergic synaptic terminals, progressive synaptic vesicle depletion and selective dopaminergic neurodegeneration—fit the pathological basis established for other truncating *DNAJC6* alleles [2, 4, 5].

### Phenotype in context: a severe multisystem presentation

Our patient’s phenotype sits at the more severe end of the published *DNAJC6* spectrum [5]. To place this presentation in context, Table 3 summarizes the principal *DNAJC6* kindreds and cases reported to date, together with their ancestry, variants, ages at onset and levodopa responses [2, 4, 5, 21–23]. Edvardson and colleagues, in the original Palestinian families, described juvenile parkinsonism with intellectual disability and seizures [2]. Köroğlu and colleagues went on to demonstrate phenotypic variability, including milder forms [4]. The larger series by Olgiati and colleagues then mapped out a continuum running from severe juvenile-onset disease—with early intellectual decline, epilepsy, dystonia, oculogyric crises and rapid neurodegeneration—through to milder early-adult-onset forms that look more like classical Parkinson disease [5]. Our patient brings nearly every severe-end feature together in a single presentation: childhood onset, progressive parkinsonism with profound bradykinesia, generalized dystonia with prominent oromandibular involvement, cognitive decline, behavioral changes, autonomic dysfunction, oculogyric crises and clinical seizures. Two further features deserve particular emphasis because they have been less consistently highlighted in earlier reports: the corticospinal signs (lower-limb hyperreflexia and bilateral extensor plantar responses), and the life-threatening cachexia visible in Fig. 1A and B. The combination of upper motor neuron signs and extrapyramidal disease in *DNAJC6*-related disease has not been a routine focus of earlier reports, and nutritional compromise of this magnitude has been under-recognized in the published phenotype.

**Table 3:** Summary of previously reported DnaJ heat shock protein family (Hsp40) member C6-related parkinsonism kindreds and cases, with the present case for comparison

Study (year) [ref.]	Ancestry/consanguinity	Affected (*n*)	*DNAJC6* variant	Onset (y)	Levodopa response and salient features
Edvardson et al. 2012 [2]	Palestinian; consanguineous	2 families	Homozygous splice-site c.801-2A > G	< 11	Juvenile parkinsonism with intellectual disability and seizures; poor or absent levodopa response
Köroğlu et al. 2013 [4]	Turkish; consanguineous	4 (1 family)	Homozygous nonsense c.2200C > T (p.Gln734Ter)	Juvenile	Parkinsonism with intellectual disability, pyramidal signs and epilepsy; phenotypic variability
Olgiati et al. 2016 [5]	Dutch and Brazilian; + sporadic	2 families + 1 sporadic	Novel homozygous variants (incl. missense p.Arg927Gly) and rare noncoding variants	3rd–5th decade	Milder, early-onset adult PD end of the spectrum; prominent levodopa response, at times limited by psychiatric side effects
Elsayed et al. 2016 [21]	Sudanese; consanguineous	1 family	Homozygous nonsense (truncating)	Juvenile	Juvenile parkinsonism with intellectual disability, pyramidal signs and epilepsy; expanded the recognized phenotype
Ng et al. 2020 [22]	Mixed cohort; several consanguineous	6 patients	Homozygous nonsense (incl. c.766C > T p.Arg256Ter)	Infancy–childhood	Neurodevelopmental delay then progressive parkinsonism-dystonia with regression and loss of ambulation; reduced/absent DaTscan uptake; largely pharmacoresistant
Garza-Brambila et al. 2021 [23]	Mexican; non-consanguineous	1	Homozygous frameshift c.2589delG	11	Juvenile dystonia-parkinsonism
Present case (2026)	Sudanese; first-cousin parents	1	Homozygous frameshift c.1480del (p.Glu494ArgfsTer12), novel	~ 5	Severe juvenile parkinsonism-dystonia with corticospinal signs, oculogyric crises, seizures and life-threatening cachexia; initial partial response with later functional gains after dose optimization; incidental BRCA1 secondary finding

Age at onset is given as reported. This table summarizes the principal reported DNAJC6 kindreds and cases and is not exhaustive. Abbreviations: *BRCA1* breast cancer 1 gene; *c.* coding DNA reference sequence (HGVS); *DaTscan* dopamine transporter single-photon emission computed tomography; *DNAJC6* DnaJ heat shock protein family (heat shock protein 40) member C6 gene; *incl.* including; *n* number of affected individuals; *p.* protein reference sequence (HGVS); *PD* Parkinson disease; *ref* reference; *y* years

### Normal brain imaging and EEG in the context of the literature

A striking feature of our patient’s workup was that both brain magnetic resonance imaging and electroencephalography were entirely normal, despite advanced multisystem disease and three clinical seizures. This is concordant with the *DNAJC6* literature, in which conventional structural neuroimaging is characteristically unremarkable—particularly in earlier disease phases—with basal-ganglia signal change and other neurodegenerative markers typically absent [2, 4, 5, 22]. Functional dopaminergic imaging, by contrast, is more informative: reduced or absent striatal tracer uptake on ^123^ iodine-123-labeled N-ω-fluoropropyl-2β-carbomethoxy-3β-(4-iodophenyl)nortropane (ioflupane [123I]) single-photon emission computed tomography (DaTscan) has been demonstrated in reported cases, although this modality was not available to us [22]. Interictal electroencephalography has likewise been reported as normal or nonspecific in *DNAJC6*-related disease even when clinical seizures occur, so a normal recording does not exclude either the diagnosis or an underlying seizure tendency [2, 4]. The practical message is that normal magnetic resonance imaging and electroencephalography should not dissuade clinicians from pursuing genetic testing in a child with a progressive hypokinetic-rigid syndrome; if anything, unremarkable structural imaging in this setting should raise, rather than lower, suspicion of a monogenic synaptic disorder.

### Diagnostic delay and the misdiagnosis of functional neurological disorder

Eight years from symptom onset to molecular diagnosis ranks among the longer delays reported in the *DNAJC6* literature. The misdiagnosis as conversion disorder, although understandable in the context of unremarkable early investigations, illustrates a recurring and consequential clinical hazard [6, 7]. Prescribing amisulpride and risperidone—both dopamine D2 receptor antagonists that actively worsen parkinsonism—to a patient with incipient dopaminergic neurodegeneration is the pharmacological nadir of that error. The clinical decline that followed was, in retrospect, predictable. The lesson is not just that the diagnosis was wrong, but that the treatment for the wrong diagnosis caused active harm. When first-line investigations are unrevealing in a child with a progressive movement disorder, particularly with consanguinity, functional neurological disorder should remain a diagnosis of rigorous exclusion, reached only after metabolic, genetic and autoimmune etiologies have been systematically considered.

### Levodopa response: from initial benefit to dose-limiting complications and later functional gains

Our patient’s levodopa response illustrates a clinically familiar trajectory in juvenile-onset monogenic parkinsonism. An initial partial improvement was not augmented by early dose escalation, and the motor benefit reached a plateau well below what would normally be expected in juvenile-onset levodopa-responsive parkinsonism. At the same time, classical levodopa-induced complications emerged—on-state dyskinesias, visual hallucinations and excessive daytime somnolence. The point is that exogenous dopamine is clearly reaching central dopaminergic circuits; she is not pharmacologically refractory in the simple sense. Rather, her residual nigrostriatal architecture cannot handle that dopaminergic load without producing aberrant motor and neuropsychiatric output [5]. Two contextual factors are likely contributory. The homozygous frameshift identified here predicts complete loss of functional auxilin protein, so the magnitude of presynaptic dopaminergic compromise may exceed that produced by missense or splice-site variants previously reported. She also went through several years of polypharmacy that included dopamine receptor antagonists, which may have contributed to lasting neuronal injury.

Importantly, her course since then argues against a fixed ceiling. When carbidopa/levodopa was cautiously up-titrated to 10/100 mg approximately every 5 hours (about five doses daily), she made a further, clinically obvious gain: she regained the ability to walk independently across a room (Additional file 5: Video 4), and her fine-motor control improved to the point that she could apply nail polish unaided (Additional file 6: Video 5)—a task that demands coordinated, sustained distal dexterity. This later response suggests that the therapeutic window, though narrow, can in some patients be widened by patient, incremental dose adjustment rather than abandoned, and that the balance between benefit and dyskinesia or neuropsychiatric toxicity is worth actively titrating over time rather than treating as fixed. In this case, *DNAJC6*-related disease behaved less as a uniformly pharmacoresistant condition and more as one in which the therapeutic window for dopaminergic replacement is narrow but genuinely exploitable with careful titration.

### Polypharmacy and the vulnerability of pediatric patients

The cluster of weakness, swallowing slowness, reduced oral intake and daytime drowsiness that emerged within days of the revised pharmacological regimen is a useful reminder of the pharmacological vulnerability of children with neurological disease exposed to multiple central nervous system-active agents at once. In a patient already operating with limited swallowing and nutritional reserves, the additive sedative load of benzodiazepines, levetiracetam, clonidine and any residual antipsychotic can produce clinically significant decompensation. The dose reductions of clonazepam and levetiracetam undertaken promptly by the supervising clinician show the appropriate clinical response; the episode also reinforces the case for cautious, sequential introduction rather than simultaneous initiation of multiple psychoactive agents wherever it is practicable.

### The BRCA1 secondary finding: clinical and ethical considerations

The incidental detection of a pathogenic heterozygous *BRCA1* variant in a patient in her early teens is clinically and ethically multidimensional [12]. The immediate personal clinical implications are limited—surveillance for *BRCA1* carriers is generally directed at adults aged 25 and older. The identification of this variant in the context of confirmed ovarian cancer in the maternal grandmother does, however, create a strong clinical case for urgent cascade testing of the patient’s mother and other at-risk female relatives [19]. If the mother is found to be a carrier, she should be enrolled immediately in an appropriate oncological surveillance program. The patient herself will need a proactive transition plan for *BRCA1*-appropriate surveillance as she approaches adulthood. The American College of Medical Genetics and Genomics position is that medically actionable secondary findings should be reported regardless of patient age; in this case, where the family history already includes an ovarian malignancy, the balance of benefit strongly favors disclosure with appropriate genetic counseling and prompt cascade action [12].

### Limitations

Several limitations deserve acknowledgement. As a single-patient report, our observations cannot be generalized without caution; the apparent narrowing of the levodopa therapeutic window may not characterize other patients with comparable loss-of-function variants. Parental carrier testing was in progress at the time of writing, and formal confirmation of biparental heterozygosity for the c.1480del variant therefore remains outstanding. The transient hepatic biochemical disturbance earlier in her course remains without a definitive mechanistic explanation. Formal neuropsychological assessment was not performed and would have added usefully to the phenotypic characterization of this novel variant. The genetic basis of her younger brother’s autism spectrum disorder has not yet been characterized, and a shared familial neurodevelopmental susceptibility cannot be excluded with certainty. The contribution—if any—of her severe perinatal respiratory compromise to her later neurological trajectory cannot be determined retrospectively. Longer-term outcome data, including any future role for deep brain stimulation, will require extended longitudinal follow-up.

## Conclusions

We have described a 13-year-old Sudanese girl in whom a novel homozygous *DNAJC6* frameshift deletion (c.1480del; p.Glu494ArgfsTer12) produced severe progressive juvenile parkinsonism with dystonia, oculogyric crises, seizures, autonomic dysfunction and life-threatening cachexia, after an 8-year diagnostic delay that included a period of misattribution to a functional neurological disorder. The case adds a previously unreported allele to the *DNAJC6* mutational catalog and extends its recognized phenotype to include corticospinal involvement and the kind of severe nutritional compromise that has not been given prominence in earlier reports. It also shows that levodopa remains useful and that careful, patient dose optimization can extend motor benefit—our patient regained independent walking and fine-motor dexterity after up-titration to 5-hourly dosing—even though dyskinesias, hallucinations and somnolence mark the ceiling of tolerability. An incidental pathogenic *BRCA1* variant identified as a medically actionable secondary finding illustrates the dual yield of clinical whole-exome sequencing and the importance of integrated counseling and family cascade pathways. Early consideration of whole-exome sequencing in childhood-onset movement disorders—especially in the presence of consanguinity—should be standard practice; functional neurological disorder must not be diagnosed by exclusion of common conditions alone, and dopamine receptor antagonists should not be prescribed before metabolic, genetic and autoimmune causes have been properly addressed.

### Patient and family perspective

*The following was prepared by the authors with the family’s knowledge and consent, as a representative paraphrase of the family’s account; the family was offered the opportunity to revise or replace the text during peer review.*

“For years, we did not know what was happening to our daughter. The doctors told us at first that her movements were ‘in her mind’, but we could see her growing weaker and more shaken by the day, and we could not believe this. The medicines made her sleepy and slower, and her body became thinner and thinner. When the genetic test finally gave a name to her illness, it was both a relief and a sadness—a relief because we could at last fight something that was real, and a sadness because there is no easy cure. She is now able to walk again and to do small things with her hands that she could not do before, and although she still needs our help with many things, these small returns mean a great deal to us. There are good moments—times when her tremor is calmer, when she laughs at something her brother says, when she eats a little more than the day before—and we hold on to those. We are grateful to the doctors who did not give up looking for an answer, and we hope our family’s story will help another family avoid the same long journey.”

## Supplementary Information

Additional file 1. Clinical videos (MP4) demonstrating the patient’s involuntary movements, including generalized tremor of mixed rest and action character with superimposed dystonic posturing affecting particularly the distal upper and lower limbs. Facial features have been anonymized.

Additional file 2. Clinical videos (MP4) demonstrating the patient’s involuntary movements, including generalized tremor of mixed rest and action character with superimposed dystonic posturing affecting particularly the distal upper and lower limbs. Facial features have been anonymized.

Additional file 3: Clinical video (MP4) demonstrating the patient’s gait, illustrating a spastic-parkinsonian pattern with slowness, reduced stride length, reduced arm swing, freezing, postural instability and right-sided predominance. The recording was obtained during a phase when assisted ambulation remained possible. Facial features have been anonymized.

Additional file 4: Whole-exome sequencing clinical report (PDF). Full molecular diagnostic report documenting the primary *DNAJC6* homozygous pathogenic frameshift finding and the secondary *BRCA1* heterozygous pathogenic finding, with ACMG/AMP variant classification rationale and laboratory accreditation details.

Additional file 5: Clinical follow-up video (MP4) recorded after carbidopa/levodopa was increased to approximately five-hourly dosing, demonstrating recovery of independent standing and walking across a room without support—a marked improvement from her earlier wheelchair dependence. Facial features have been anonymized.

Additional file 6: Clinical follow-up video (MP4) recorded after the same dose optimization, demonstrating improved distal upper-limb dexterity, with the patient able to apply nail polish unaided. Facial features have been anonymized.

Additional file 7: (CARE checklist). Populated CARE (CAse REport) checklist completed in accordance with JMCR submission requirements.

## Data Availability

No datasets were generated or analyzed during the current study.

## Abbreviations

ACMG: American College of Medical Genetics and Genomics
ALP: Alkaline phosphatase
ALT: Alanine aminotransferase
AMP: Association for Molecular Pathology
AR: Autosomal recessive
AST: Aspartate aminotransferase
BRCA1: Breast cancer 1 gene
CARE: CAse REport (reporting guideline)
CT: Computed tomography
DaTscan: Dopamine transporter single-photon emission computed tomography
DBS: Dried blood spot
DNAJC6: DnaJ heat shock protein family (Hsp40) member C6
EEG: Electroencephalogram/electroencephalography
GGT: Gamma-glutamyl transferase
gnomAD: Genome Aggregation Database
GRCh38: Genome Reference Consortium Human Build 38
HBOC: Hereditary breast and ovarian cancer
HSG: Hysterosalpingography
MRC: Medical Research Council (motor power grading)
MRI: Magnetic resonance imaging
NBIA: Neurodegeneration with brain iron accumulation
NICU: Neonatal intensive care unit
NMD: Nonsense-mediated mRNA decay
ORCID: Open Researcher and Contributor ID
PD: Parkinson disease
PEG: Percutaneous endoscopic gastrostomy
SSPE: Subacute sclerosing panencephalitis
VUS: Variant of uncertain significance
WES: Whole-exome sequencing
