# Supplementary material for: Childhood-onset parkinsonism, dystonia and severe muscle wasting in a Sudanese girl caused by a novel homozygous DnaJ heat shock protein family (heat shock protein 40) member C6 frameshift variant, with an incidental pathogenic breast cancer 1 gene secondary finding: a case report
Source: J Med Case Rep. 2026 Jul 22;20:639. doi: 10.1186/s13256-026-06351-x (PMC13679260; doi:10.1186/s13256-026-06351-x)
Supplement: Supplementary file 1 [file 13256_2026_6351_MOESM4_ESM.pdf]

# 3B-EXOME, Proband

Clinical use

Unique ID: [redacted]
3billion ID: EPL25-RTTI

## PATIENT INFORMATION

| | | | | | |
|---|---|---|---|---|---|
| **Unique ID** | [redacted] | **Physician** | Delta laboratories | **Sample type** | DBS |
| **3billion ID** | EPL25-RTTI | **Department** | Medical genetics | **Collected on** | 2025-12-01 |
| **DOB* / Sex** | 2012-12-24 / Female | **Institution** | Delta medical laboratory | **Ordered on** | 2025-12-07 |
| **Ethnicity** | African/african-american | | | **Accessioned on** | 2025-12-11 |

* (YYYY-MM-DD)

## CLINICAL INFORMATION

| | |
|---|---|
| **Symptoms** | Neurological abnormality, Muscle spasm, Inability to walk, Inability to speak<br>(Additional Notes provided have been reviewed and utilized for the result interpretation. You can find the Additional Notes in the Method section below.) |

## RESULT SUMMARY

| | | | |
|---|---|---|---|
| **Primary finding** | Variant reported | **Additional findings** | No variant reported |
| **Secondary finding** | Variant reported | | |

Analysis and interpretation were conducted for various genetic variants, including single nucleotide variants, small insertions/deletions, copy number variants, mitochondrial variants, repeat expansions, and structural variants. See below the Methods section for limitations.

## PRIMARY FINDINGS

# POSITIVE

A homozygous pathogenic variant was identified in *DNAJC6*. *DNAJC6* is associated with autosomal recessive '*DNAJC6*-related Parkinson disease (OMIM: 608375)'. As this variant has never been reported in other patients, clinical correlation are recommended. Parental testing is also recommended to confirm if parents are carriers of the variant.

***DNAJC6*-related Parkinson disease** (OMIM: 608375)

| Gene | Variant | Classification |
|---|---|---|
| *DNAJC6* | **Genomic Position:** 1-65392440-CG-C (GRCh38) | Pathogenic |
| | **cDNA:** NM_001256864.2:c.1480del | |
| | **Protein:** NP_001243793.1:p.Glu494ArgfsTer12 | |
| | **Zygosity:** Homozygous | |
| | **Inheritance:** Unknown | |

# 3B-EXOME, Proband

Clinical use

Unique ID: [redacted]
[redacted]
3billion ID: EPL25-RTTI

## PRIMARY FINDINGS INTERPRETATION

| *DNAJC6* NM_001256864.2:c.1480del (NP_001243793.1:p.Glu494ArgfsTer12) | |
|---|---|
| **Population Data** | The variant is not observed in the gnomAD v4.1.0 dataset. |
| **Predicted Consequence/Location** | Frameshift: predicted to result in a loss or disruption of normal protein function through nonsense-mediated decay (NMD) or protein truncation. Multiple pathogenic variants are reported downstream of the variant. |
| **Segregation Data** | Regardless of the mechanism, a variant called homozygous is by default *in trans*. |
| **Computation and Functional Data** | None |
| **Previously Reported Variant Data** | None |
| **Disease Association** | *DNAJC6*-related Parkinson disease (OMIM: 608375) |
| **Validation** | Not performed as the variant was considered high-quality |
| **Variant Classification** | Pathogenic |

## ADDITIONAL FINDINGS

No additional variants were identified, including variants of uncertain significance (VUSs) that could not be reported as primary findings due to limited evidence of pathogenicity, even though they may explain the patient's symptoms; pathogenic, likely pathogenic variants or VUSs that may partially explain the patient's symptoms, regardless of whether they fit the mode of inheritance; or variants associated with the family history provided by the healthcare provider, regardless of the patient's current symptoms.

# 3B-EXOME, Proband

Clinical use

Unique ID: [redacted]

3billion ID: EPL25-RTTI

## SECONDARY FINDINGS

A heterozygous pathogenic variant was identified in *BRCA1* that is associated with 'Breast-ovarian cancer, familial, 1 (OMIM: 604370)'. *BRCA1* is part of the 84 medically actionable secondary finding genelist established by the American College of Medical Genetics and Genomics (ACMG). Breast-ovarian cancer, familial 1 (OMIM: 604370) is an autosomal dominant, multifactorial disorder. Individuals with pathogenic variants in *BRCA1* (OMIM: 113705) have an increased risk for developing breast cancer and ovarian cancer (includes fallopian tube and primary peritoneal cancers) and other cancers such as prostate cancer, pancreatic cancer, and melanoma to a lesser extent. Genetic counseling and clinical management are warranted.

| Breast-ovarian cancer, familial, 1 (OMIM: 604370) | | |
|---|---|---|
| **Gene** | **Variant** | **Classification** |
| *BRCA1* | **Genomic Position:** 17-43091488-CCT-C (GRCh38) | Pathogenic |
| | **cDNA:** NM_007294.4:c.4041_4042del | |
| | **Protein:** NP_009225.1:p.Gly1348AsnfsTer7 | |
| | **Zygosity:** Heterozygous | |
| | **Inheritance:** Unknown | |

## RESOURCES

- Online Mendelian Inheritance in Man®:This report contains information from the Online Mendelian Inheritance in Man® (OMIM®) database, which has been obtained under a license from Johns Hopkins University. This report does not represent the entire, unmodified OMIM® database, which is available in its entirety at http://omim.org/downloads.
- gnomAD (genome Aggregation Database): gnomad.broadinstitute.org
- ClinVar (National Center for Biotechnology Information ClinVar Database): ncbi.nlm.nih.gov/clinvar
- HGVS (Human Genome Variation Society): varnomen.hgvs.org
- HGMD (The Human Gene Mutation Database) Professional
- MITOMAP (A human mitochondrial genome database): https://www.mitomap.org/MITOMAP

## REFERENCES

1. Richards S et al. ACMG Laboratory Quality Assurance Committee. Standards and guidelines for the interpretation of sequence variants: a joint consensus recommendation of the American College of Medical Genetics and Genomics and the Association for Molecular Pathology. Genet Med. 2015 May;17(5):405-24. PMID: 25741868.
2. Erin R et al. Technical standards for the interpretation and reporting of constitutional copy-number variants: a joint consensus recommendation of the American College of Medical Genetics and Genomics (ACMG) and the Clinical Genome Resource (ClinGen). Genet Med. 2020 Feb;22(2):245-257.
3. Elizabeth M et al. Specifications of the ACMG/AMP standards and guidelines for mitochondrial DNA variant interpretation. Hum Mutat. 2020 Dec;41(12):2028-2057.
4. Seo GH et al. Diagnostic yield and clinical utility of whole exome sequencing using an automated variant prioritization system, EVIDENCE. Clin Genet. 2020 Dec;98(6):562-570. PMID: 32901917.
5. Lee, K., Abul-Husn, N.S., Amendola, L.M. et al. ACMG SF v3.3 list for reporting of secondary findings in clinical exome and genome sequencing: A policy statement of the American College of Medical Genetics and Genomics (ACMG). Genet Med. 2025 un 23;27(8):101454 PMID 40568962.
6. Dhong-Gun Won et al. 3Cnet: pathogenicity prediction of human variants using multitask learning with evolutionary constraints. Bioinformatics. 2021 Jul 16;btab529. PMID: 34270679.
7. McKenna A, Hanna M, Banks E, Sivachenko A. et al. The Genome Analysis Toolkit: a MapReduce framework for analyzing next-generation DNA sequencing data. Genome Res. 2010 Sep;20(9):1297–303. PMID: 20644199
8. Xiaoyu Chen, Ole Schulz-Trieglaff, Richard Shaw, et al. Manta v1.6.0: rapid detection of structural variants and indels for germline and cancer sequencing applications. Bioinformatics. 2016 Apr 15;32(8):1220-2. PMID: 26647377
9. Dolzhenko E, Deshpande V, Schlesinger F, et al. ExpansionHunter: a sequence-graph-based tool to analyze variation in short tandem repeat regions. Bioinformatics. 2019 Nov 15;35(22):4754–6. PMID: 31134279
10. Gardner EJ, Lam VK, Harris DN, et al. The Mobile Element Locator Tool (MELT): population-scale mobile element discovery and biology. Genome Res. 2017 Nov;27(11):1916–29. PMID: 28855259
11. Quinodoz M, Peter VG, Bedoni N, et al. AutoMap is a high performance homozygosity mapping tool using next-generation sequencing data. Nat Commun. 2021 Jan 22;12(1):518. PMID: 33483490

# 3B-EXOME, Proband Clinical use

## NOTES

1. Results summary: Results are categorized into positive, inconclusive and negative

| Category | Explanation |
|---|---|
| **Positive** | • AD or XL disease: one heterozygous or hemizygous P/LP variant is identified in a known disease gene.<br>• AR disease: one homozygous P/LP variant or two P/LP (potential) compound heterozygous variants are identified in a known disease gene. |
| **Inconclusive** | • AD or XL disease: one heterozygous or hemizygous VUS is identified in a known disease gene.<br>• AR disease: At least two heterozygous or one homozygous VUS are identified in a known disease gene.<br>• AR disease: One heterozygous P/LP variant is identified in a known disease gene.<br>• A P/LP variant(s) are identified in a GUS that has sufficient evidence of being a disease gene. |
| **Negative** | • No clinically significant variant that would fit the patient's phenotype well is identified. |

Abbreviation: AD; autosomal dominant, AR; autosomal recessive, XL; X-linked, P; Pathogenic, LP; likely pathogenic, VUS; variant of uncertain significance, GUS; gene of uncertain significance.

2. Variant Classification: A variant is classified according to the ACMG guideline (PMID 25741868) using the type of evidence including population data, computational and predictive data, functional data, segregation data, de novo data, and allelic data.

## RECOMMENDATIONS

1. Genetic counseling is warranted to review the test results and interpretation.
2. This test can detect single nucleotide variants, small insertions/deletions (<50 bp) and large (>=3 consecutive exons) copy number variants with high accuracy in most of the genomic regions. If low level (<20%) mosaicism variants on autosomes and sex chromosomes, small (<3 consecutive exons) copy number variants (CNVs), structural variants (SVs) including inversions and translocations, or low heteroplasmic level mitochondrial genome variants are suspected, it is recommended to perform other tests specifically designed to detect these types of variants. Variants in regions of high sequence homology, such as pseudogenes, may be difficult to detect. Intronic variants, epigenetic factors, or variants in regulatory regions called by being near coding exons may not be interpretable.
3. The test results are based on the clinical information and family history provided in the test order. If the information provided is incorrect or insufficient, the test may not yield reliable results. If the test results have weak clinical correlations, additional testing may be required at the discretion of your medical provider. Whole exome sequencing test or Sanger sequencing test on the biological parents or other family members is recommended to confirm segregation of the variant(s). For structural variants (SVs), including copy number variants (CNVs), only variants for which the exact breakpoint has been identified can be tested by Sanger sequencing. Low level heteroplasmic (<20%) mitochondrial variants cannot be tested by Sanger sequencing.
4. Variant interpretation is based on currently available scientific and medical information that were publicly available at the time the results were reported. Therefore, the referenced data may not be current at the time of genetic counseling.
5. In case of a negative result with no significant variants reported, it does not rule out the possibility of having a genetic condition. As new clinical/scientific information becomes available, variant classification may change and a new diagnosis can emerge. If the patient has consented to reanalysis, 3billion will perform an automated daily reanalysis using the most recent information and send an updated report to the ordering physician if the results change. The provider may also add new phenotypic information about the patient. To comply with the Korean Bioethics and Safety Act 53 and the operational procedures set forth by 3billion, Inc., specimens will be retained for a maximum period of six months and disposed of without prior notice thereafter. Consequently, any genetic variants identified and reported through reanalysis will not be subject to confirmation by Sanger sequencing unless the patient provides a new specimen.
6. Patient consent for reanalysis is renewed on a 10-year cycle. After 10 years, when a physician renews the patient's consent for reanalysis, the reanalysis period will be extended for another 10 years.

## METHODS

Genomic DNA was extracted from DBS specimen using standard protocol. Exome capture was performed using xGen Exome Research Panel v2, supplemented with xGen human mtDNA panel and xGen Custom Hyb Panel v2 (Integrated DNA Technologies, Coralville, Iowa, USA). Sequencing was performed using NovaSeq X (Illumina, San Diego, CA, USA). In total, 13,426,656,505 bases of sequence were generated and uniquely aligned to a modified version of the Genome Reference Consortium Human Build 38 (GRCh38), in which falsely duplicated regions of chromosome 21 were masked to N's based on the masking file developed in collaboration with the GRC (Genome Reference Consortium) (Nat Biotechnol. 2022;40:672-680) and Revised Cambridge Reference Sequence (rCRS) of the mitochondrial genome, generating 204.87 mean depth-of-coverage within the 35,392,153 bases of the captured region, which is approximately 99.3% of the RefSeq protein coding region. Approximately 98.70% of the targeted bases were covered to a depth of ≥20x. Despite the insufficient coverage across 1.30% of the bases (see below for details), these metrics are consistent with high quality exome sequencing data and deemed adequate for analysis. Gene or exon level depth-of-coverage (DOC) information is available upon request. In total, 71,029 single nucleotide variants (SNV) and 13,118 small insertions and deletions (INDEL) were identified. Sequencing data analysis and variant interpretation were performed using 3billion's proprietary system, EVIDENCE v4.3 (Clin Genet. 2020;98:562-570). EVIDENCE incorporates bioinformatics pipeline for calling SNV/INDEL based on the GATK best practices (GATK v4.4.0, Genome Res. 2010;20:1297–303) and Manta v1.6.0 (Bioinformatics. 2016;32:1220-2) for calling CNV (copy number variants) based on paired-end information and 3bCNV v2.1, an internally developed tool, for calling CNV (copy number variants) including aneuploidy based on the DOC information. It also incorporates Mutect2 v4.4.0 (Genome Res. 2010;20:1297–303) for calling lower level heteroplasmic SNV/INDEL in the mitochondrial genome, ExpansionHunter v5.0.0 (Bioinformatics. 2019;35:4754–6) for calling repeat expansion variants, MELT v2.2.2 (Genome Res. 2017;27:1916–29) for calling mobile element insertion variants, AutoMap v1.3 (Nat Commun. 2021;12:518) for detecting regions of homozygosity (ROH). Variant Effect Predictor v112.0 (VEP, Ensembl, Genome Biology 2016;17:122) is used for variant annotation. Variants were prioritized based on the guideline recommended by the American College of Medical Genetics and Genomics (ACMG) and the Association for Molecular Pathology (AMP) (Genet Med. 2015;17:405-424, Genet Med. 2020:22:245-257, and Hum Mutat. 2020;41:2028-2057) in the context of the patient's phenotype, relevant family history and previous test results provided by the ordering physician. Only variants deemed clinically significant and relevant to the patient's clinical indications at the time of variant interpretation are reported. Based on internal studies validating the accuracy of the variants called with high quality scores, only low quality variants are confirmed by Sanger sequencing. The raw data files including FASTQ files, VCF files and/or annotated small variant lists are available upon request.

## ADDITIONAL NOTE

The patient presents with recurrent neurological episodes characterized by muscle spasms, generalized body tremors, and intermittent inability to walk or speak. Despite extensive evaluation—including comprehensive laboratory testing and brain MRI—the results have been unremarkable, and no definitive diagnosis has been established. Given the persistent unexplained neurological symptoms and the negative findings from routine investigations, Whole Exome Sequencing (WES) is recommended to identify a potential underlying genetic etiology.

# 3B-EXOME, Proband

Clinical use

Unique ID[redacted]

3billion ID: EPL25-RTTI

## DISCLAIMER

This test was developed by 3billion in the purpose of identifying single nucleotide variants (SNV), small insertions/deletions (INDEL, <50 bp), large (>=3 consecutive exons) copy number variants, mobile element insertion variants and repeat expansion variants within the targeted genomic regions. Repeat expansion detection is possible for the following 18 genes. Repeat expansion number may be underestimated for the starred (*) gene with compromised sensitivity (*AR, ARX, ATN1, ATXN1, ATXN2, ATXN3, ATXN7, ATXN8OS*, CACNA1A, COMP, FOXL2, HOXD13, HTT, PABPN1, PHOX2B, PRDM12, TBP, ZIC2*). Only SNV/INDEL (>10% heteroplasmic level) are called within the mitochondrial genome. This test is intended for clinical purposes and should not be regarded as investigational or for research. This laboratory is certified under the College of American Pathologists (CAP#:8750906) and Clinical Laboratory Improvement Amendments (CLIA#: 99D2274041) as qualified to perform high complexity clinical laboratory testing. Assay validation and clinical validation were performed following the Korea Institute of Genetic Testing Evaluation and the American College of Medical Genetics and Genomics (ACMG) Technical Standards and Guidelines Section G (https://www.acmg.net/PDFLibrary/Standards-Guidelines-Clinical-Molecular-Genetics.pdf). If other types of variants such as translocation, inversion, low-level mosaicism, low heteroplasmic level mitochondrial genome variants, and mitochondrial genome large deletion/duplication are suspected, it is recommended to perform appropriate testing that are designed to detect those types of variants. Also, there are certain exonic regions that are incompletely sequenced due to technical difficulties with amplification, sequencing and alignment. If variants within these regions are suspected, it is recommended to perform alternate testing that are designed to sequence those regions/genes adequately. This report may not be copied or reproduced, except in its totality.

## ACCREDITATIONS AND CERTIFICATIONS

**CAP License #**
8750906, AU-ID# 2052626

**CLIA ID #**
99D2274041

This case has been comprehensively reviewed by our clinical team of physicians, geneticists and informaticists.

Report electronically signed by:

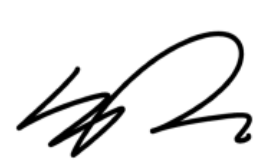

**Seung Woo Ryu, Ph.D.**
Clinical Genomics Scientist

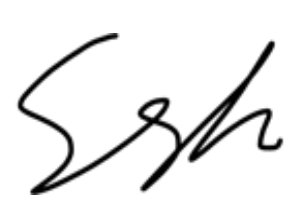

**Go Hun Seo, M.D, Ph.D.**
Chief Medical Officer & Laboratory Director
