# Supplementary material for: Childhood-onset parkinsonism, dystonia and severe muscle wasting in a Sudanese girl caused by a novel homozygous DnaJ heat shock protein family (heat shock protein 40) member C6 frameshift variant, with an incidental pathogenic breast cancer 1 gene secondary finding: a case report
Source: J Med Case Rep. 2026 Jul 22;20:639. doi: 10.1186/s13256-026-06351-x (PMC13679260; doi:10.1186/s13256-026-06351-x)
Supplement: Supplementary file 2 [file 13256_2026_6351_MOESM7_ESM.docx]

**CARE Checklist — populated**

**Additional file 5 — populated CARE (CAse REport) checklist**

**Manuscript:** Childhood-onset parkinsonism, dystonia and severe muscle wasting caused by a novel homozygous *DNAJC6* frameshift variant, with an incidental pathogenic *BRCA1* secondary finding: A case report.

**Submitted to:** *Journal of Medical Case Reports* (BMC / Springer Nature).

**Corresponding author:** Khabab Abbasher Hussien Mohamed Ahmed, Faculty of Medicine, University of Khartoum, Khartoum 11111, Sudan. Email: khabab9722@gmail.com. ORCID: 0000-0003-4608-5321.

**CARE statement:** This case report was prepared in accordance with the CARE (CAse REport) reporting guideline (Gagnier JJ, Kienle G, Altman DG, Moher D, Sox H, Riley D, et al. The CARE guidelines: consensus-based clinical case reporting guideline development. *J Med Case Rep*. 2013;7:223; *http://www.care-statement.org/*). All items in the checklist below have been addressed in the manuscript at the locations indicated.

| **Topic** | **Item** | **Checklist item description** | **Reported on (section / location)** |
| --- | --- | --- | --- |
| Title | **1** | The diagnosis or intervention of primary focus followed by the words "case report". | Title page — title ends in ": A case report". |
| Key Words | **2** | 2 to 5 key words that identify diagnoses or interventions in this case report, including "case report". | Keywords section (after Abstract): DNAJC6; juvenile parkinsonism; dystonia; BRCA1; whole-exome sequencing; secondary findings; conversion disorder; oculogyric crises; consanguinity; case report. [Note: 10 keywords given to meet JMCR's 3–10 keyword requirement; all CARE-recommended key terms included.] |
| **Abstract (no references)** | | | |
|  | **3a** | Introduction: What is unique about this case and what does it add to the scientific literature? | Abstract — Background paragraph (novel homozygous DNAJC6 frameshift; eight-year diagnostic delay; incidental BRCA1 finding). |
|  | **3b** | Main symptoms and/or important clinical findings. | Abstract — Case presentation paragraph (tremor onset at age 5; progression to parkinsonism, dystonia, cachexia, oculogyric crises, seizures, wheelchair dependence). |
|  | **3c** | The main diagnoses, therapeutic interventions, and outcomes. | Abstract — Case presentation paragraph (WES diagnosis; levodopa response; dose-limiting complications). |
|  | **3d** | Conclusion — What is the main "take-away" lesson(s) from this case? | Abstract — Conclusions paragraph (novel allele; severe phenotype; harm from FND misdiagnosis; early WES in childhood movement disorders). |
| Introduction | **4** | One or two paragraphs summarizing why this case is unique (may include references). | Background section — three paragraphs covering monogenic juvenile PD, DNAJC6/auxilin biology, the diagnostic hazard of FND misattribution, and what this case adds. References [1–7]. |
| **Patient Information** | | | |
|  | **5a** | De-identified patient specific information. | Case presentation — Demographics and family background ("A 13-year-old Sudanese girl of African ethnicity, presently resident in the Middle East…"). Patient name, medical record number and date of birth are not included anywhere in the manuscript. |
|  | **5b** | Primary concerns and symptoms of the patient. | Case presentation — Symptom evolution (chronological narrative of tremor onset, progression, multisystem features). |
|  | **5c** | Medical, family, and psycho-social history including relevant genetic information. | Case presentation — Demographics and family background (consanguinity, family epilepsy, maternal grandmother ovarian cancer, brother autism); Perinatal and developmental history (HSG-assisted conception workup, prematurity, NICU course, normal milestones); Investigations → Whole-exome sequencing (DNAJC6 primary finding; BRCA1 secondary finding). |
|  | **5d** | Relevant past interventions with outcomes. | Case presentation — Diagnostic pathway and misdiagnosis as functional neurological disorder (initial polypharmacy regimen including fluoxetine, carbamazepine, amisulpride, trihexyphenidyl, risperidone, co-careldopa; partial improvement followed by deterioration; medications discontinued pending re-evaluation). |
| Clinical Findings | **6** | Describe significant physical examination (PE) and important clinical findings. | Case presentation — General examination; Neurological examination (cranial nerves; motor tone and power; reflexes; sensation; coordination; involuntary movements; gait). Detailed MRC motor power grading in Table 1. Figure 1 (A and B) showing muscle wasting. |
| Timeline | **7** | Historical and current information from this episode of care organized as a timeline. | Case presentation — Symptom evolution and Diagnostic pathway sub-sections, structured as a chronological narrative timeline (age 5 onset → mid-childhood progression → age ~11 wheelchair dependence → FND misdiagnosis with polypharmacy → genetic referral → WES diagnosis → revised treatment → adverse event and dose adjustment → current state). A separate timeline figure can be added during peer-review if required. |
| **Diagnostic Assessment** | | | |
|  | **8a** | Diagnostic testing (such as PE, laboratory testing, imaging, surveys). | Case presentation — Investigations → Laboratory studies (TFTs, FBC, iron studies, magnesium, vitamin B1); Liver function (markedly abnormal initially → normalised on retesting); Imaging and electrophysiology (MRI, CT, EEG); Whole-exome sequencing (methods and platform). Comprehensive summary in Table 2. |
|  | **8b** | Diagnostic challenges (such as access to testing, financial, or cultural). | Background (paragraph 3) flags the broader pitfall. Case presentation — Diagnostic pathway and misdiagnosis as FND describes the specific challenges encountered (unrevealing first-line investigations leading to a psychiatric label; subsequent harm from D2-receptor antagonist exposure; modest socioeconomic circumstances; eight-year delay to molecular diagnosis). Further discussed in Discussion → "Diagnostic delay and the misdiagnosis of functional neurological disorder". |
|  | **8c** | Diagnosis (including other diagnoses considered). | Case presentation — Differential diagnosis section (Wilson disease, Charcot-Marie-Tooth disease, Rett syndrome, SSPE, NBIA, autoimmune encephalitis, functional neurological disorder, each addressed in turn with rationale for exclusion). Final diagnosis confirmed in Investigations → Whole-exome sequencing → Primary finding (DNAJC6 c.1480del homozygous pathogenic). |
|  | **8d** | Prognosis (such as staging in oncology) where applicable. | Case presentation — Outcome and follow-up; Discussion → Phenotype in context (severe end of DNAJC6 spectrum); Discussion → Levodopa response: from initial benefit to dose-limiting complications. No formal staging system exists for DNAJC6-related disease; prognosis is addressed narratively. |
| **Therapeutic Intervention** | | | |
|  | **9a** | Types of therapeutic intervention (such as pharmacologic, surgical, preventive, self-care). | Case presentation — Treatment section (pharmacological: co-careldopa, baclofen, clonazepam; transient adjuncts: olanzapine, clonidine, levetiracetam). Multidisciplinary supportive care: physiotherapy, occupational therapy, speech and swallowing evaluation, nutritional rehabilitation (specialist dietitian, planned NG/PEG as required), genetic counselling. |
|  | **9b** | Administration of therapeutic intervention (such as dosage, strength, duration). | Case presentation — Treatment (specific dosing reported: levodopa/carbidopa 10/100 mg four times daily; baclofen 5–10 mg per dose titrated; clonidine 25 µg at night; durations and dose adjustments described). |
|  | **9c** | Changes in therapeutic intervention (with rationale). | Case presentation — Treatment section describes: (i) initial regimen and partial response; (ii) revised regimen following expert specialist opinion (addition of clonidine, levetiracetam; olanzapine taper for incompatible dopamine antagonism); (iii) emergent polypharmacy-related decompensation and the resulting clonazepam and levetiracetam dose reductions; (iv) current regimen at most recent review with rationale for narrow therapeutic window. |
| **Follow-up and Outcomes** | | | |
|  | **10a** | Clinician and patient-assessed outcomes (if available). | Case presentation — Outcome and follow-up (clinician-assessed: persistent wheelchair dependence, progressive cognitive decline, modest motor improvement on dopaminergic therapy). Patient and family perspective section (family-assessed outcomes: described in the family's own voice). |
|  | **10b** | Important follow-up diagnostic and other test results. | Case presentation — Investigations → Liver function — repeat testing (full normalisation, supporting exclusion of Wilson disease). Outcome and follow-up describes ongoing parental genetic testing for DNAJC6 c.1480del and cascade BRCA1 testing in progress. |
|  | **10c** | Intervention adherence and tolerability (How was this assessed?). | Case presentation — Treatment (tolerability assessed at clinical review; family-reported adherence; supervising clinician monitored emergent adverse effects in real time). Outcome and follow-up — Levodopa-induced complications subsection details on-state dyskinesias, hallucinations and somnolence that now constrain further dose escalation. |
|  | **10d** | Adverse and unanticipated events. | Case presentation — Diagnostic pathway (deterioration following dopamine antagonist exposure during the FND-directed regimen); Treatment (acute polypharmacy-attributable weakness, swallowing slowing, drowsiness, with prompt dose reductions); Outcome — Levodopa-induced complications (dyskinesias, visual hallucinations, excessive daytime somnolence). |
| **Discussion** | | | |
|  | **11a** | A scientific discussion of the strengths AND limitations associated with this case report. | Discussion and conclusions → Limitations sub-section (single-patient generalisability; outstanding parental carrier confirmation; unexplained transient hepatic disturbance; absence of formal neuropsychological assessment; brother's ASD genetic basis uncharacterised; perinatal contribution undeterminable; need for longer follow-up). Strengths embedded in opening paragraph and throughout (variant novelty; multisystem phenotypic characterisation; documented dual yield from clinical WES). |
|  | **11b** | Discussion of the relevant medical literature with references. | Discussion and conclusions sub-sections (Molecular pathology of c.1480del; Phenotype in context — comparison with Edvardson, Köroğlu and Olgiati cohorts [refs 2,4,5]; Diagnostic delay and misdiagnosis of FND [refs 6,7]; Levodopa response [refs 5,17]; Polypharmacy vulnerability; BRCA1 secondary finding [refs 12,19]). All 20 references cited with first-appearance numbering. |
|  | **11c** | The scientific rationale for any conclusions (including assessment of possible causes). | Discussion → Molecular pathology of the c.1480del variant (NMD-mediated loss of auxilin function with downstream synaptic and dopaminergic consequences) provides the molecular rationale. Discussion → Levodopa response addresses pharmacological rationale for the narrow therapeutic window. Limitations explicitly acknowledge alternative explanations. |
|  | **11d** | The primary "take-away" lessons of this case report (without references) in a one paragraph conclusion. | Discussion and conclusions → Conclusions sub-section (single paragraph): novel allele; phenotypic extension; narrow levodopa therapeutic window; importance of dual WES yield; early WES in childhood-onset movement disorders; avoid premature FND labelling and pre-genetic D2-receptor antagonist exposure. |
| Patient Perspective | **12** | The patient should share their perspective in one to two paragraphs on the treatment(s) they received. | Patient and family perspective section (single paragraph in the family's own paraphrased voice). The family was offered the opportunity to revise or replace this text during peer review. [Note: the patient herself has cognitive impairment that limits direct first-person narration; the family's perspective is reported on her behalf with their explicit consent.] |
| Informed Consent | **13** | Did the patient give informed consent? Please provide if requested. | YES. Written informed consent for publication of the case report, including clinical details, laboratory and genetic data, anonymised photographs and video recordings, was obtained from the patient's parent / legal guardian (the patient is a minor). The signed consent form is held by the corresponding author and is available for inspection by the Editor-in-Chief on request. Documented in Declarations → Consent for publication. |

**Author signature and date**

Khabab Abbasher Hussien Mohamed Ahmed (corresponding author): ___________________________ Date: _____16/06/2026_____

CARE checklist source: Riley DS, Barber MS, Kienle GS, Aronson JK, von Schoen-Angerer T, Tugwell P, et al. CARE guidelines for case reports: explanation and elaboration document. *J Clin Epidemiol*. 2017;89:218–35. Checklist available at http://www.care-statement.org/.
